# Dual blockade of IL-17A and IL-36 pathways via a bispecific antibody exhibits enhanced anti-inflammatory potency

**DOI:** 10.3389/fimmu.2024.1434127

**Published:** 2024-11-12

**Authors:** Xiaojuan Ma, Shuang Zhang, Xiaochen Ren, Yujie Feng, Hui Li, Shi Chen, Jingen Xu, Yanting Wang, Guo-yuan Peng, Qingran Yan, Huifeng Jia, Simin Xia, Xiaopei Cui, Xiaofang Chen, Xianfei Pan, Shaojie Wang, Haijia Yu, Xiaoyue Wei, Mingwei Li, Bei Liu, Jingyue Xu, Qiaoxia Qian, Xiangyang Zhu, Yifan Zhan, Liangjing Lu

**Affiliations:** ^1^ Rheumatology, Shanghai Jiao Tong University School of Medicine Affiliated Renji Hospital, Shanghai, China; ^2^ Department of Drug Discovery, Shanghai Huaota Biopharmaceutical Co. Ltd., Shanghai, China; ^3^ Department of Pharmacology, School of Pharmacy, Fudan University, Shanghai, China

**Keywords:** bispecific antibody, IL-17A, IL-36R, skin inflammation, skin fibrosis

## Abstract

Antibody drugs targeting single inflammatory cytokines have revolutionized the treatment of immune-mediated inflammatory diseases. To investigate whether dual targeting interleukin-17 (IL-17) and IL-36 enhances anti-inflammatory activity, bispecific Ab HB0043 was generated by linking the single chain fragment variables (scFvs) from humanized anti-IL-36R antibody (HB0034) to the C-terminus of the heavy chain of anti-IL-17A IgG1 (HB0017) Fc using a flexible peptide linker. HB0043 largely maintained the binding affinities and biological activities of the two parent monoclonal antibodies (mAbs) *in vitro*. IL-17 and IL-36 cooperated to amplify the expression of pro-inflammatory and pro-fibrotic genes in normal human dermal fibroblasts (NHDF). However, HB0043 more effectively blocked IL-6 and IL-8 production in NHDF stimulated by IL-17A and IL-36 compared to two monoclonal antibodies. In a mouse model of Oxazolone (OXA)-induced atopic dermatitis and Imiquimod (IMQ)-induced skin inflammation, administration of both anti-IL17A mAb HB0017 and anti-mouse IL-36R surrogate antibody HB0034SA showed improved effectiveness in alleviating skin thickening and inflammation based on histological assessment. Further, in cynomolgus monkeys, HB0043 showed no enhanced target-related toxicity compared with the two parental mAbs *in vivo* and with a moderate increase in production of anti-drug antibodies. Together, dual blockade of IL-17A and IL-36R in the form of a bispecific antibody may have advantages in blocking the overlapping and non-overlapping functions of these two cytokines in skin inflammation that could not optimally be curtailed with single mAbs. In conclusion, as monotherapy may reach therapeutic celling for certain difficult-to-treat inflammatory and fibrotic diseases, dual targeting could potentially pave a way to combat these diseases.

## Highlights

- A novel bispecific antibody (BsAb) targeting IL-17A and IL-36R maintained functional activity comparable to that of its two parental monoclonal antibodies (mAbs).
- The BsAb outperformed its parental mAbs in suppressing the secretion of IL-6 and IL-8 induced by co-stimulation with IL-36 and IL-17A.
- Dual target therapy showed improved effectiveness in alleviating skin thickening and inflammation in a mouse model of oxazolone-induced dermatitis.
- The BsAb showed no enhanced target-related toxicity compared with the two parental mAbs *in vivo*.

## Introduction

Cytokines play a key role in acute and chronic inflammation, local tissue injury, and immune regulation (1, 2). Therefore, cytokines are closely related to the pathogenesis of various inflammatory diseases and can be used as potential targets for disease treatment (3–7). Although blocking single cytokine signaling has a significant effect in some inflammatory diseases, it has only a weak effect in others (8). Blockade of multiple cytokines has previously been investigated in select disease types. For example, dual blockade of IL-4 and IL-13 is required to broadly inhibit type 2 inflammation (9) and in a mouse model of collagen-induced arthritis, mice blocking both TNF and IL-17 had milder disease compared to those of blocking each cytokine alone (10). Thus, dual target biologic therapy may broaden the treatment for inflammatory diseases.

Interleukin-17A (IL-17A) is a main pro-inflammatory factor of the IL-17 family that plays an important role in the pathogenesis of skin inflammation (11, 12). IL-17A is mainly secreted by helper T cell 17 (Th17 cell), a subset of CD4^+^ T cells preferentially producing IL-17A, IL-17F, IL-21, and IL-22. IL-17A can also be produced by many other cell populations including CD8^+^ (Tc17) cells, γδT cells, natural killer T (NKT) cells, group 3 innate lymphoid cells (ILC3s), and MAIT cells (13, 14). The results of clinical trials show that IL-17 antagonists are effective in moderate-to-severe plaque psoriasis (15–17), psoriatic arthritis (PsA) (18, 19) and ankylosing spondylitis (AS) (20–22). Nevertheless, the effectiveness of IL-17 antagonism alone in treatment of other inflammatory diseases such as lupus nephritis (NCT04181762) was less obvious. Of note, the simultaneous neutralization of both IL-17A and IL-17F more effectively suppressed production of inflammatory mediators compared to the inhibition of IL-17A or IL-17F alone (23). In addition to being an inflammatory activator itself, IL-17A also has a strong synergistic effect with other inflammatory effectors (24–26). Notably, in cultured primary human keratinocytes (KC), IL-17A synergizes with IL-22 and tumor necrosis factor (TNF)-α to induce the expression of IL-36, a cytokine with critical involvement in skin inflammation (27). IL-36 is a member of the IL-1 superfamily, composed of three agonists IL-36α, IL-36β, IL-36γ and two antagonist IL-36Ra and IL-38 (28–30). Furthermore, IL-36 agonists promote the proliferation of CD4^+^ T cells with IL-36R expression, which in turn increases the secretion of IFN-γ, IL-4, and IL-17A (31). Mutations in the IL-36RN gene, encoding the IL-36Ra protein, are closely associated with the development of generalized pustular psoriasis (GPP) (32–34). Spesolimab, IL-36 receptor inhibitor, is approved for the treatment of GPP. Beyond acute inflammatory GPP, IL-36 has also been implicated in fibrosis of multiple tissues/organs including skin (35). Notably, IL-36R antagonist alone in treatment of palmoplantar pustulosis and atopic dermatitis had limited effectiveness (36). It raises a question whether monotherapy may reach therapeutic celling for certain difficult-to-treat inflammatory and fibrotic diseases and whether dual targeting could potentially enhance the effectiveness of treatment by blocking multiple pathways of inflammatory responses. As both IL-17 and IL-36-mediated inflammatory axis play a critical role in skin inflammation, evident by successful treatment of their corresponding skin inflammatory diseases and interplay of two cytokines in skin inflammation (37–39), we envisage that dual blockade of IL-17A and IL-36R could have enhanced potency to disrupt the IL-17 and IL-36 inflammatory loop to broaden the treatment indications for both targets. To this end, we designed a novel bispecific antibody HB0043, which targets both IL-36R and IL-17A. *In vitro* characterization of HB0043 had indicated that it was superior to its two parent monoclonal antibodies in inhibiting the inflammatory action of IL-36 and IL-17A. *In vivo* investigation with surrogate Abs in a skin inflammatory model also demonstrated that dual blockade alleviated inflammation with enhanced potency. Together, we believe that dual target therapy with biologics might increase the potential clinical benefit to treat skin diseases and more.

## Materials and methods

### Generation of humanized anti-human IL-17A mAb, anti-human IL-36R and anti-mouse IL-36R mAbs

Generation and characterization of humanized anti-human IL-17A mAb HB0017 has been described previously (40, 41). Animal use for generation of humanized anti-human IL-36R mAb HB0034 and anti-mouse IL-36R mAb had approvals of IACUC of Shanghai Model Organisms. For generation of humanized anti-human IL-36R mAb HB0034, BALB/c mice were immunized with recombinant human IL-36R protein (ACRO Biosystems, Cat# IL2-H5254). Mice with high titers detected by ELISA assay were sacrificed. Harvested mouse spleen cells were fused with SP2/0 myeloma cells to generate hybridomas according to standard methods. The hybridomas with strong binding and blocking activity to human IL-36R and monkey IL-36R, but not cross-react to IL1R1, were selected for further characterization. The murine antibody genes from selected monoclonal hybridomas were sequenced, cloned and then humanized by grafting CDR into human homologous framework. The humanized antibody variable genes were cloned into expression vectors containing constant regions of human IgG1 heavy chain and kappa light chain. For the preparation of antibody-dependent cell-mediated cytotoxicity (ADCC) silent antibody, the Fc leucines at 234 and 235 (EU numbering) in the CH2 region of the HB0017 Fc domain were mutated into two alanine (L234A/L235A) to eliminate antibody’s binding affinity to FcγR and C1 to abolish ADCC and complement dependent cytotoxicity (CDC) activities. For generation of surrogate anti-mouse IL-36R mAb HB0034SA, IL-36R knockout mice on C57BL/6 background (Shanghai Model Organisms Co., Ltd) were immunized with mouse IL-36R protein (R&D Biosystems, Cat# 2354-RP/CF) emulsified in complete Freund’s adjuvant. Harvested spleen cells from immunized mice with high antibody titers were fused with SP2/0 myeloma cells to generate hybridomas according to standard methods. Hybridomas with strong binding and blocking activity to mouse IL-36R were selected for production of HB0034SA.

### Engineering of IgG-scFv bispecific antibodies, protein expression, and purification

Humanized anti-IL-36R mAb was chosen as the scFv component of bispecific Abs. The scFv was in VL → VH orientation, with a (GGGGS)n (n=3,4,5) linker (linker 2) connecting the VL to the VH. Anti-IL-36R scFvs were linked to the C-terminus of the heavy chain of anti-IL-17A IgG1 Fc using a flexible peptide linker (G4S)4(linker 1). The DNA sequences were synthesized (Genewiz) and subcloned into pHBG vector (prepared in house) and amplified in DH5α competent cells. Purified plasmids after linearization (50 μg in total, heave chain: light chain = 1:2) were then transfected into CHO-K1 cells (Chinese Hamster Ovary cells) to develop stable cell lines using the electro-transformation technique. Selected monoclonal cell line, based yield and protein stability, was then used for production of BsAb proteins used for preclinical studies.

For the purification of BsAb proteins, the cells were cultured in a shaker for seed expansion until the viable cell density of cell lines achieved up to 1 × 10^6^ cells/mL. Cells were then cultured in the bioreactors for about 15 days in a fed-batch mode. The antibodies were captured from the cell supernatants using protein A resin (MabSelect SuRe, Cytiva), and then purified by anion exchange chromatography (Capto Adhere, Cytiva) and cation exchange chromatography (Poros 50HS, Thermo Fisher). The generated antibodies were then evaluated for purity, aggregation, charge heterogeneity and protein glycosylation modification before used for preclinical studies.

### Enzyme-linked immunosorbent assay for binding activity

Binding ability of mAbs and BsAbs was measured with a designed ELISA assay. Antigen (Human IL-36R, ACRO Biosystems, Cat# IL2-H52H6; Mouse IL-36R, R&D Biosystems, Cat# 2354-RP/CF) diluted with carbonate buffer and further coated on 96 well-plate overnight (2-8°C). Block the plate with 3% defatted Milk (w/v)-0.05% (v/v) Tween20-PBS buffer and serial diluted samples (range from 0.079 pM to 333 nM) was added and then the plate was incubated for 1 hour at room temperature. Thereafter, goat anti-human IgG-HRP (Thermo Fisher, Cat# A18817) and goat anti-mouse IgG (Jackson ImmunoResearch, Cat# 115-035-164) corresponding to the sample Fc and incubate at room temperature for 1 hour. The substrate solution was added for 10 min before adding stopping solution (1M H_2_SO_4_). Absorbance was measured using microplate reader (SpectraMax i3Max, Molecular Devices, US) at 450 nm wavelength. 4-parameter logistic curve was plotted with antibody concentration against absorbance.

### Surface plasma resonance assay

All SPR experiments were performed at 25°C, 10 Hz with a Biacore™ 8K System (Cytiva, Sweden), and sensorgrams were analyzed with the Biacore Insight Evaluation Software. The samples were prepared in HBS-EP+ buffer, pH7.4 (Cytiva, Cat# BR-1006-69), which constituted the running buffer. To determine IL-17A binding kinetic, CM5 sensor chip (Cytiva, Cat# 29149603) was immobilized with anti-human Fc antibody (Cytiva, Cat# 26234600). HB0017 or HB0043 were captured on sensor chip by a 1 min pulse at a flow rate of 10 μL/min. Analytes (IL-17A, ranging from 0.06 to 5 nM) were injected for 120 s, and dissociation was measured for 600 s at a rate of 50 μL/min. Surface regeneration was achieved with a 30 s pulse of 3M MgCl_2_ (Cytiva, Cat# 26234600).

To assess the binding affinity of antibodies to IL-36R, antigen (IL-36R) was captured on a CM5 sensor chip (Cytiva, Cat# 29149603) immobilized with anti-6×His tag antibody (Cytiva, Cat# 28995056) as ligand. Analytes (HB0034 or HB0043, ranging from 0.78 to 100 nM) were injected for 120 s, and dissociation was measured for 1200 s at a rate of 50 μL/min. Surface regeneration was achieved with a 90 s pulse of 10 mM glycine (pH1.5) (Cytiva, Cat# BR-1003-54).

### Cell culture

Cells were grown and maintained at 5% CO_2_ at 37°C. NCI/ADR-RES cells purchased from Cobioer (China; Cat# CBP60380R; lot# 5B67) were grown and maintained in MEM (Gibco, Cat# 11095080) supplemented with 10% FBS (Gibco, Cat# 10099141C), 1% MEM Non-Essential Amino Acids Solution (NEAA, Gibco, Cat# 11140050), 1 mM Sodium Pyruvate (Gibco, Cat# 11360070) and 1 μM Adriamycin (Selleck, Cat# S1208). NIH-3T3 cells purchased from ATCC (Cat# CRL-1658; lot# P70035213) were grown and maintained in DMEM (Gibco, Cat# 11995065) supplemented with 1% NBCS (Gibco, Cat# 16010159). HT1080 cells were purchased from ATCC (Cat# CCL-121; lot# FF44) and cultured with 10% FBS + MEM (Gibco, Cat# 11095080).

### Functional blocking assay

For blocking Human IL-36R, NCI/ADR-RES cells (human ovarian cancer *cells*) were seeded in a 96-well plate in 100 μL volume at 4.5 × 10^5^ cells/mL and incubate overnight. IL-36 cytokine and serial diluted mAb/BsAb were premixed and further added into plate. Then the plate was incubated for 4 h. IL-6 level in the harvested supernatants were quantified using ELISA kit (Cat#430501, Biolegend) as per manufacturer’s instructions. For blocking Mouse IL-36R: NIH-3T3 cells (mouse NIH/Swiss embryo fibroblast cells) were seeded in a 96-well plate in 100 μL volume at 3 × 10^5^ cells/mL and incubate overnight. IL-36β (85 pM, Novoprotein, China; Cat#CK25) and serial diluted mAb/BsAb were added into plate, then incubate for 24 hours. CXCL1 level in the harvested supernatants were quantified using ELISA kit (Cat# 447504, Biolegend) as per manufacturer’s instructions. For blocking human IL-17A: HT1080 cells (human fibrosarcoma cells) were seeded in a 96-well plate in 50 μL volume at 2.0 × 10^4^ cells/well. The mixture of 10 ng/mL human IL-17A (Sino Biological, Cat#12047-HNAS), 10 ng/mL human TNF-α (Sino Biological, Cat#10602-HNAE) and serial diluted mAb/BsAb were added to the wells with a final volume of 200 μL/well. Incubate for 24 hours. IL-6 levels in the harvested supernatants were quantified using ELISA kit (Cat# 430501, Biolegend) as per manufacturer’s instructions.

### RNA sequencing

NHDF (normal human dermal fibroblasts) (Promocell, Cat# C-12302; lot# 0FFA) in DMEM (Gibco, Cat# 10566016) supplemented with 10% FBS and 1% Penicillin-Streptomycin (Gibco, Cat# 15140122) were seeded at 2 × 10^6^ cells/mL in 1 mL volume in a 6-well plate and incubated overnight. Cells were then stimulated with IL-36 ligands (α/β/γ each at 30 ng/ml, R&D, Cat#6995-IL-MTO, 6834-ILB-025, 6835-IL-010), human IL-17A (200ng/mL, R&D, Cat# 7955-IL-025/CF) or both IL-36 ligands and IL-17A for 24 hours. Harvested cells was collected for RNA extraction. RNA purification, reverse transcription, library construction and sequencing were then performed at Shanghai Majorbio Bio-pharm Biotechnology Co., Ltd. (Shanghai, China) according to the manufacturer’s instruction (Illumina, San Diego, CA). The NHDF RNA-seq transcriptome library was prepared following Illumina® Stranded mRNA Prep, Ligation from Illumina (San Diego, CA) using 1 μg of total RNA.

### Reverse transcription polymerase chain reaction

NHDF were diluted to 2 × 10^6^ cells/mL in culture medium DMEM (Gibco, Cat# 10566016) + 10% FBS + 1% Penicillin-Streptomyci (P/S, Gibco, Cat# 15140122) and seeded in a 6-well plate with 1 mL/well. After overnight incubation in the incubator containing 5% CO_2_ at 37°C, cells were stimulated with human IL-17A (100 ng/ml), IL-36 ligand mixtures or both for 24 hours. Harvested cells were then subjected to RNA purification. RNAs were then used to perform RT-PCR using Rneasy Micro Kit (Qiagen, Cat# 74004), PrimeScript™ RT reagent Kit with gDNA Eraser (Perfect Real Time) (Taraka, Cat# RR047A) and Fast Start Universal SYBR Green Master (Rox) (Roche, Cat# 04913914001) according to the manufacturers’ instructions. cDNA was amplified by PCR using primers for human GAPDH as housekeeping gene.

The primer sequences:

**Table:** 

Primers	Sequences (5′- 3′)
IL-6-F	AGCCCACCGGGAACGAAA
IL-6-R	CCGAAGGCGCTTGTGGAG
IL-8-F	GAAGTTTTTGAAGAGGGCTGAGA
IL-8-R	TGCTTGAAGTTTCACTGGCATC
CCL2-F	TCTCGCCTCCAGCATGAAAG
CCL2-R	GGCATTGATTGCATCTGGCT
CCL20-F	GCGAATCAGAAGCAAGCAAC
CCL20-R	GCATTGATGTCACAGCCTTCA
CXCL2-F	AAGCTTGTCTCAACCCCGC
CXCL2-R	CTGGTCAGTTGGATTTGCCATTTT
MMP3-F	GGACAAAGGATACAACAGGGACC
MMP3-R	GAACCGAGTCAGGTCTGTGAG
MMP9-F	GATCATTCCTCAGTGCCGGA
MMP9-R	GTTCAGGGCGAGGACCATAG
GAPDH-F	GGAGTCCACTGGCGTCTTCA
GAPDH-R	GGGGTGCTAAGCAGTTGGTG

### Assessment of functional blocking activity of HB0043

For the evaluation of functional potency of HB0043, NHDF (3 × 10^4^ cells/well in 100 μL) were incubated in 96-well plates with serial diluted Abs (HB0034, HB0017 or HB0043) in the presence of 0.5 ng/ml of IL-17A plus 15 ng/ml of IL-36α, 1ng/ml IL-36β or 2 ng/ml IL-36γ in final volume of 200 μL for 24 hrs. After overnight culture, supernatant was collected and assayed for IL-6 and IL-8 with ELISA kits (Biolegend, Cat# 430515/431501) following manufacture instruction.

### Ethics statement

Animal studies were carried out in compliance with the recommendations in the Guidelines on the Care and Use of Animals for Scientific Purposes of the National Advisory Committee for Laboratory Animal Research. Mice studies had received ethic approvals from IACUC of HkeyBio (Suzhou) Technology Co. Ltd. and Weiyuan (Suzhou) Technology Co. Ltd. For pharmacokinetic and toxicology analysis in cynomolgus monkeys, Ethic approvals were gained from the institutional animal care and use committees of Joinn Laboratories (Suzhou) Inc; GuangXi Fangcheng Gang Spring Biological Technology Development Corporation Ltd.; Guangzhou Huazhen Biotechnology Co., Ltd., IACUC at West-China-Frontier PharmaTech Co., Ltd.

### Oxazolone-induced mouse atopic dermatitis model

C57BL/6J mice purchased from Vital River (male, 7-to 9-week-old) had their back hair shaved using a hair trimmer and their skin depilated with 0.5-g Veet hair removal gel 1 day before the application of OXA (Macklin, Cat# E904804) in acetone (Yonghua, Cat# 43202). 5.0% OXA was applied on the shaved back skin and ear of the mice on Day 0. A daily topical dose of 5.0% OXA was then applied on the shaved back skin and 0.5% OXA on the ear of the mice for twenty-one consecutive days from Day 7 for acute disease induction.

As HB0034 did not cross-react to murine IL-36R, a surrogate mAb, HB0034SA, which specifically targeted murine IL-36R and blocked signaling of mouse IL-36 agonists was used instead. Anti-IL-17A antibody HB0017 used in this study, which forms the backbone of bispecific Ab HB0043, cross-reacts to mouse IL-17A (40). In acute OXA-induced skin inflammation, mice were intraperitoneally administered either HB0017 (twice a week, 50mg/kg) or HB0034SA (twice a week, 50mg/kg) or in combination of both (twice a week, 50 + 50 mg/kg). The control group received PBS intraperitoneal injections.

The severity of inflammation was measured every two days using Severity Index. Oedema, erythema, dryness and excoriation were scored independently on a scale from 0 to 3: 0, none; 1, slight; 2, moderate; 3, severe. The measurements of right ear thickness and the evaluation of the clinical scores of the back skin were performed once every two days. At the end of study, the back skin at the hair removal area was harvested. Skin sections after H&E staining were analyzed for the pathological phenotype. Histomorphometry was performed using an OsteoMeasure image analysis software program (OsteoMetrics, Inc., Atlanta, GA) interfaced with an Olympus light/epifluorescent microscope and video subsystem.

### IMQ-induced mouse psoriasis model

BALB/c mice purchased from Charles River (male, 6-week-old) had their back hair shaved using a hair trimmer 1 day before. 5.0% IMQ (62.5mg) was applied on the shaved back skin of the mice daily, from Day 1 to Day 7 for acute disease induction. Mice were intraperitoneally administered either HB0017 (twice a week, 50mg/kg) or HB0034SA (twice a week, 50mg/kg) or in combination of both. The control group received PBS intraperitoneal injections.

The severity of inflammation was measured using Severity Index. Oedema, erythema, dryness and excoriation were scored independently on a scale from 0 to 4: 0, none; 1, slight; 2, moderate; 3, severe; 4, extremely severe. The evaluation of the clinical scores of the back skin were performed once every day. At the end of study, the back skin at the hair removal area was harvested for section and qPCR. Skin sections after H&E staining were analyzed for the pathological phenotype. Histomorphometry was performed using an OsteoMeasure image analysis software program interfaced with an Olympus light/epifluorescent microscope and video subsystem. Skin section (*n=8*/group) were independently scored for stratum corneum, epidermis, dermis by a pathologist in a blind fashion with the following criteria: Stratum corneum: hyperplastic corneas (0.5); Epidermis: thinning of nipples (0.5), trochanterellus lengthen and rodlike (1.5), acanthosis (0.5); Dermis: lymphocytes infiltration (2.0), papillarispars congestion (0.5); Total score is the sum of stratum corneum score, epidermis score and dermis score. Treatment and genotypes were not disclosed to investigators performing the histology and generating semi-quantitative readouts.

### Pharmacokinetic and toxicology

Eighteen cynomolgus monkeys were assigned to 3 groups (3/sex), administered HB0017 (3, 10, or 30 mg/kg), HB0034 (5, 15, or 50 mg/kg) and HB0043 (3, 10, or 30 mg/kg) once via subcutaneous or intravenous injection, respectively. After dosing, approximately 1.5 ml blood sample was collected from the hindlimb at different time points. The serum was separated by centrifuge. The drug concentrations in serum were determined. Pharmacokinetic parameters were determined using non-compartmental analysis (NCA) in WinNonlin.

For toxicology analysis, forty cynomolgus monkeys were assigned to four groups, 5 animals/sex/group and were administered HB0017 at 15, 50, or 150 mg/kg/dose once per week for 4 weeks via subcutaneous injection, HB0034 at 50, 100, or 250 mg/kg/dose once per week for 4 weeks via intravenous infusion, respectively. 0.9% saline or 20, 100, or 200 mg/kg/dose HB0043 once per week for 4 weeks via intravenous infusion, followed by a 6-week recovery. Assessment of toxicity was based on mortality, clinical observations, body weights, food consumption, ophthalmic observations, dose site observations, body temperatures, blood oxygen, blood pressure, immune globulin, electrocardiographic (ECG) measurements, clinical pathology and anatomic pathology.

### Statistical analyses

Statistical analyses were performed using GraphPad Prism v10.2 (GraphPad Software, La Jolla, CA). Statistical significance between control and experimental groups was analyzed by one-way ANOVA or indicated in the figure legends. P values were corrected for multiple testing according to Tukey when several conditions were compared with each other within one experiment. The criterion for statistical significance was P<0.05.

## Results

### A tetravalent bispecific antibody targeting IL-17A and IL-36R had anti-IL-17A IgG1 backbone with anti-IL-36R single-chain variable fragments

**Figure 1:**
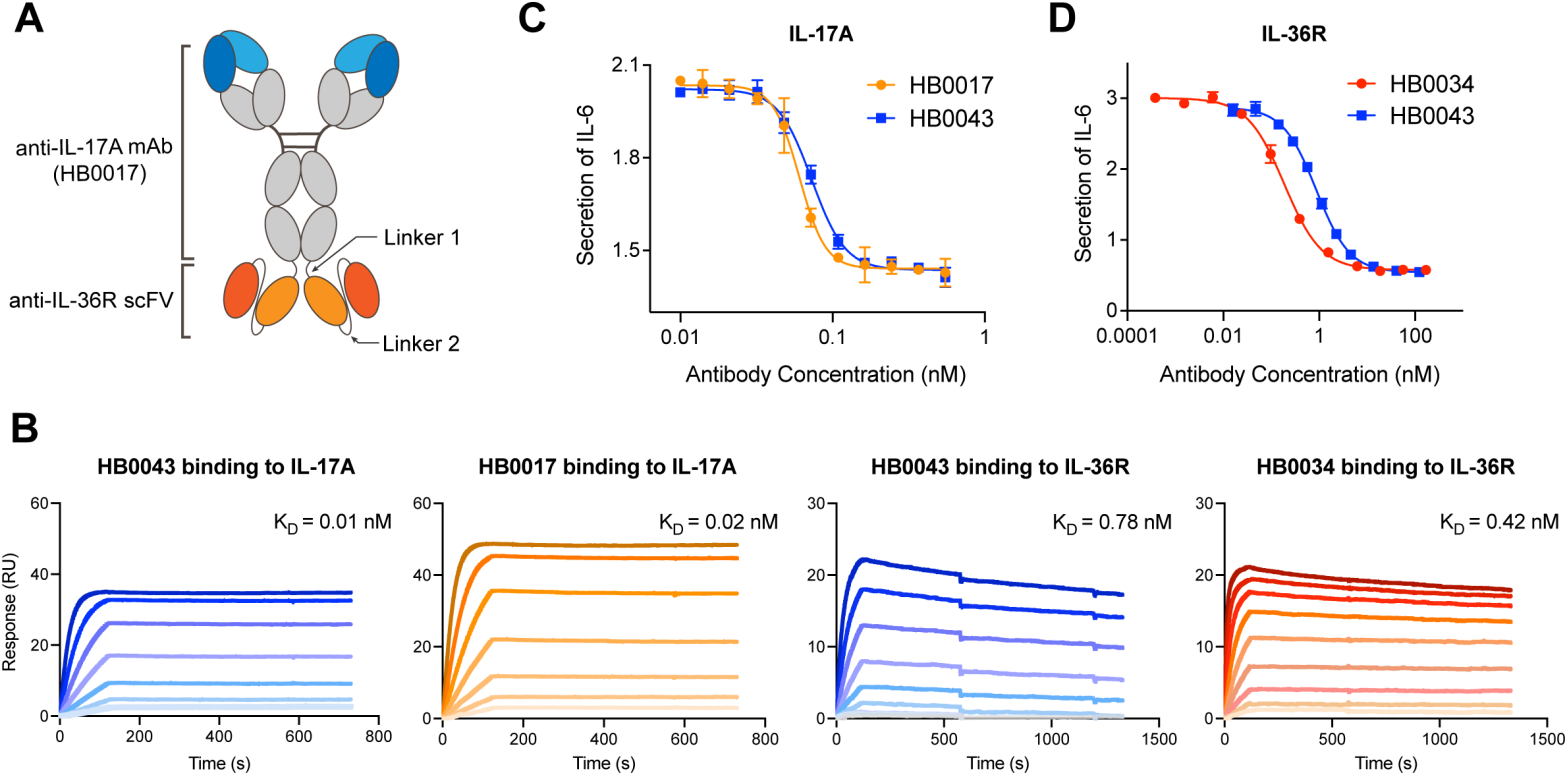
HB0043 retained the affinities and blockade activity to both targets. **(A)** Schematics of the anti-IL-36R×IL-17A BsAb (HB0043) structure. HB0043 was constructed by linking a scFv targeting IL-36R to the C-terminal domain of the anti-IL-17A IgG backbone. **(B)** The kinetics profile of HB0043 and HB0017 (the parental anti-IL-17A mAb) binding to human IL-17A, and human IL-36R binding to HB0043 and HB0034 (the parental anti-IL-36R mAb) measured by SPR assay. **(C)** The ability of HB0043 to neutralize IL-17A signaling was measured and compared to that of HB0017, using an IL-17A blockade cell-based assay. The secretion of IL-6 was monitored, which was induced by human IL-17A (0.3 nM) and TNF-α (0.6 nM) in HT-1080 cell. **(D)** The inhibition of IL-6 releases from NCI/ADR-RES cells was used as an evaluation of IL-36R signaling inhibition. NCI/ADR-RES cells were stimulated with human IL36α (13.5 nM) in the presence of HB0043 (32 pM to 245 nM) and HB0034 (4 pM to 336 nM).

The anti-human IL-17A and anti-human IL-36R BsAb was constructed by genetically linking the tandem HB0034 (anti-IL-36R antibody) single-chain variable fragments (scFvs) to the C-terminus of the heavy chain (HC) of HB0017 (anti-IL-17A antibody) as the format of immunoglobulin (Ig) G-scFv (**Figure 1A**). HB0017 had shown efficacy in various inflammatory disease models and Phase Ib trial in patients with moderate-to-severe plaque psoriasis (40, 41). The purity, thermal stability and functional activity of three BsAb candidates with different length were compared to evaluate the effects of length of linker 2. All these three BsAbs exhibited similar potent functional blockade on IL-36 signaling in NCI/ADR-RES cells and IL-17 signaling in HT1080 cells (**Table 1**). The purity and stability of 700009 showed was better than that of 700005 and 700007 (**Table 1**), Clone 700009 was therefore chosen as the final candidate and named HB0043.

**Table 1:** Structural features, SEC purity and thermodynamic properties of anti-IL-36R×IL-17A BsAbs.

Anti-IL-36R×IL-17A BsAbs	700005	700007	700009 (HB0043)
Length of linker 2	15	20	25
SEC purity(%)	86.4	91.9	94.5
Tm value (°C)	67.8	67.7	68.3
Tagg value (°C)	61.6	60.7	61.8
NCI/ADR-RES IC50 (nM)	0.5	0.5	0.7
HT1080 IC50 (nM)	0.1	0.1	0.1

### HB0043 retained the binding activity to IL-17A and IL-36R, neutralizing activity against IL-17A and blocking activity against IL-36R

To confirm the influence of binding and blocking activity of the two targets after construction as a BsAb, we compared the affinity and function of HB0043 to the two parental monoclonal antibodies (mAbs), respectively. The SPR analysis (BIAcore) results showed HB0043 can bind IL-17A with high affinity (0.01 nM). This was comparable to that of HB0017, which was 0.02 nM (**Figure 1B** and **Table 2**). These data suggest that the presence of the scFv on the C-terminus of the heavy chain of HB0017 did not affect binding of BsAb to IL-17A. Similarly, there was only a 2-fold change between the affinity KD value of HB0043 (0.78 nM) and HB0034 (0.42 nM) binding IL-36R (**Figure 1B** and **Table 2**).

**Table 2:** Binding affinity of BsAbs and mAbs to the targets from different species.

Antibody	IL-36R	ka (M^-1^s^-1^)	kd (s^-1^)	KD (nM)
HB0043	Human	3.91×10^5^	3.04×10^-4^	0.78
	Cynomolgus	2.13×10^5^	2.56×10^-3^	12.00
	Rabbit	NB	NB	NB
	Rat	NB	NB	NB
	Mouse	NB	NB	NB
HB0034	Human	1.69×10^6^	7.06×10^-4^	0.42
	Cynomolgus	4.66×10^5^	6.82×10^-3^	14.60
	Rabbit	NB	NB	NB
	Rat	NB	NB	NB
	Mouse	NB	NB	NB
HB0034SA	Human	NB	NB	NB
	Rabbit	NB	NB	NB
	Rat	1.05×10^6^	1.70×10^-4^	0.16
	Mouse	7.04×10^5^	4.00×10^-4^	0.57
	IL-17A	ka (M^-1^s^-1^)	kd (s^-1^)	KD (nM)
HB0043	Human	1.29×10^7^	1.41×10^-4^	0.01
	Cynomolgus	4.03×10^6^	5.35×10^-4^	0.13
	Rabbit	9.68×10^5^	3.54×10^-3^	3.66
	Rat	3.63×10^5^	1.65×10^-3^	4.54
	Mouse	1.44×10^6^	7.10×10^-4^	0.49
HB0017	Human	1.50×10^7^	2.34×10^-4^	0.02
	Cynomolgus	4.87×10^6^	5.14×10^-4^	0.11
	Rabbit	2.25×10^6^	8.51×10^-3^	3.78
	Rat	1.66×10^5^	1.25×10^-3^	7.50
	Mouse	5.37×10^6^	1.03×10^-3^	0.19

NB, No Binding.

In addition to binding activity, we also assessed the blocking activity of the BsAb in cell-based assays. As expected, the HB0043 was indeed capable of neutralizing IL-17A and blocking IL-36R. To assess the bioactivity of BsAb against IL-17A, HT1080 cells were stimulated by co-incubation with IL-17A (0.3 nM) and TNF-α (0.6 nM) in the presence of various concentrations of antibodies. HB0043 showed dose-dependent neutralization of IL-17A with similar activity of the anti-IL-17A mAb HB0017. The IC50 (the antibody concentration required for 50% of maximum inhibition) of HB0043 inhibiting IL-6 secretion in HT1080 cells was 0.07 nM, which was comparable to the IC50 of HB0017 (0.06 nM, **Figure 1C**). Similarly, the HB0043 effectively inhibited the secretion of IL-6 from NCI/ADR-RES cells stimulated by IL-36α (13.5 nM) at an IC50 of 0.87 nM, which was 5 times weaker than that of HB0034 whose IC50 was 0.19 nM (**Figure 1D**). These data demonstrated that the ability of HB0043 to bind and neutralize IL-17A is comparable to the parent monoclonal antibody, while the activity of blocking the IL-36R pathway is weaker than that of HB0034 *in vitro*.

### IL-17A and IL-36 additively induced IL-6 and IL-8 secretion in NHDF

**Figure 2:**
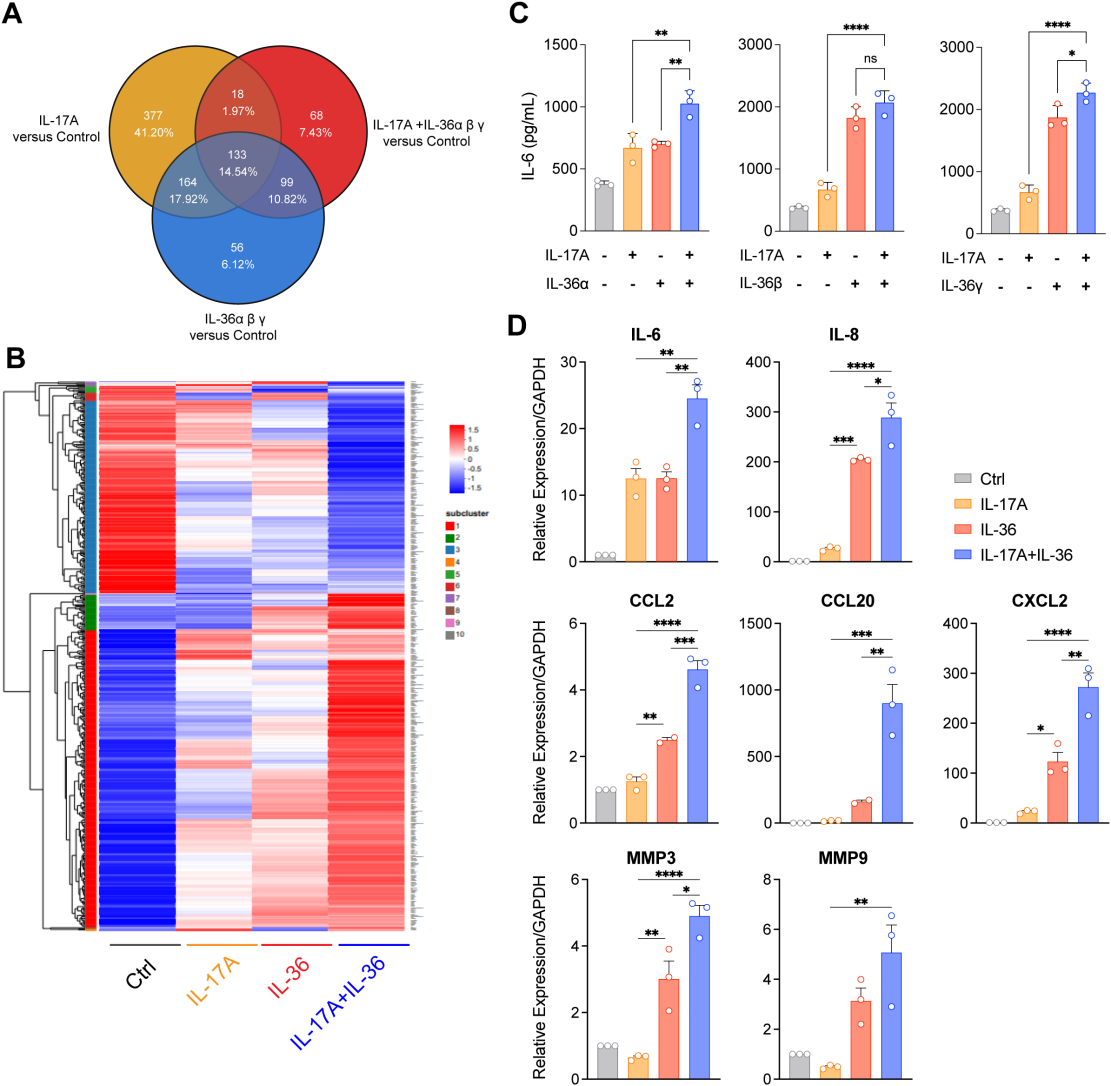
IL-36 synergized with IL-17A to promote inflammatory cytokines expression in NHDF. **(A, B)** RNA sequencing was performed for NHDF cultured with human IL-17A (100 ng/ml), IL-36 ligand (IL-36α 30 ng/ml + IL-36β 30 ng/ml + IL-36γ 30 ng/ml), and IL-36 ligand (IL-36α 30 ng/ml + IL-36β 30 ng/ml + IL-36γ 30 ng/ml) plus IL-17A (100 ng/ml) proteins for 24 hours. Differential gene expression profiling (FCH ≥ 2, FDR < 0.05). FCH, fold change; FDR, false discovery rate. **(A)** Venn diagram displaying the number and overlap of differentially expressed genes compared with those of the control. **(B)** Hierarchically clustered heatmap of inflammatory related differential gene expression of these groups. **(C)** The synergy effect of IL-17A and IL-36 ligand in the induction of IL-6 secretion. NHDF cells were cultured with 0.5 ng/ml IL-17A and 15 ng/ml IL-36α, 1 ng/ml IL-36β or 2 ng/ml IL-36γ overnight. **(D)** Various gene expression was analyzed by RT-PCR in NHDF cell following stimulation by IL-17A, IL-36 or IL-17A&IL-36 combined. Results demonstrated the synergistic effect of IL-17A and IL-36 in inducing inflammatory related gene expression. *P<0.05, **P<0.01, ***P<0.01, ****P<0.0001, ns, no significance.

To explore the effects of IL-17A and IL-36, alone or in combination, on the expression of inflammation-related genes and to screen out genes that could be used as antibody efficacy evaluation criteria, the transcriptional responses of NHDF stimulated by IL-17A and IL-36 were tested by RNA sequencing. Comparative analysis of the transcriptional responses showed that combinational stimulation with IL-36 and IL-17 displayed differential gene expression, compared to control, IL-36 or IL-17A alone (**Figures 2A, B**). Further, several differential expressed genes including cytokines, chemokines and fibrosis-related genes were selected for confirmation by RT-qPCR. Indeed, IL-6, IL-8, CCL20, CCL2, MMP3 and MMP9 were induced by stimulation with IL-36 or IL-17A alone (**Figure 2D**). Notably, combinational stimulation with IL-36 and IL-17 induced further upregulation of these genes compared to single stimulation (**Figure 2C**). Finally, released cytokine levels were also evaluated. Fittingly, levels of IL-6 (**Figure 2C**) and IL-8 (**Supplementary Figure 1A**) were higher in groups with combinational stimulation.

### HB0043 potently blocked secretion of IL-6 and IL-8 by NHDF stimulated with IL-36 and IL-17A

We next compared the inhibitory effect of HB0043, HB0017 and HB0034 on the production of IL-6 and IL-8 secretion in NHDF induced with IL-17A and IL-36 ligands. To the end, serial diluted HB0017, HB0034 or HB0043 were added into cell cultures before adding the mixtures of IL-17A and IL-36 ligands. Production of IL-6 and IL-8 in harvested culture supernatants were determined.

**Figure 3:**
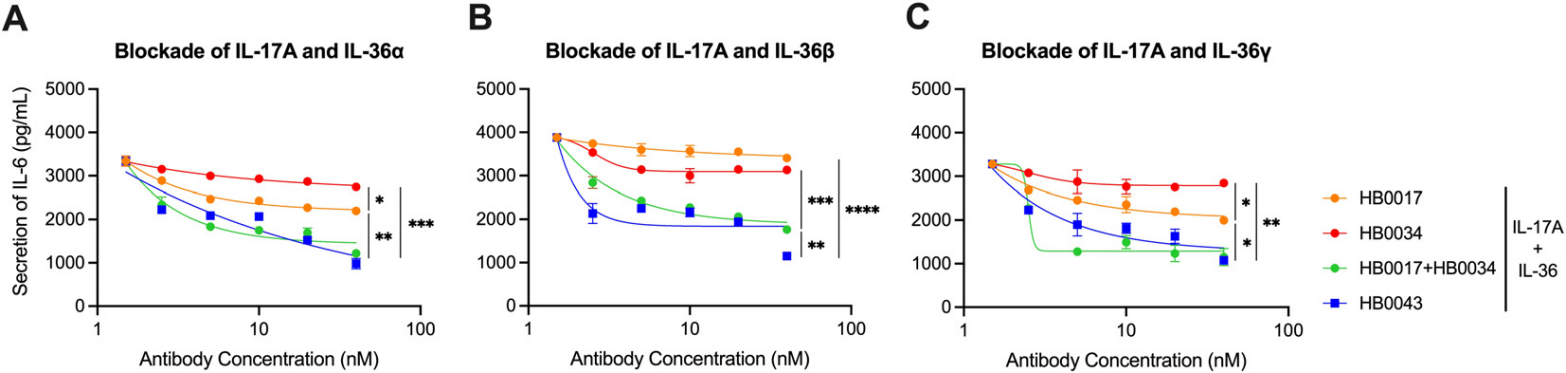
HB0043 effectively antagonized the production of IL-6 induced by IL-17A and IL-36 in NHDF cells. **(A–C)** The ability of HB0043 to block IL-36R signaling and IL-17A signaling was measured and compared to that of HB0034 and HB0017, using IL-17A and IL-36R blockade cell-based assay. The secretion of IL-6 was monitored, which was induced by 0.5 ng/ml human IL-17A and 15 ng/ml IL-36α **(A)**, 1 ng/ml IL-36β **(B)** or 2 ng/ml IL-36γ **(C)** in NHDF cells overnight. Data are expressed as means ± SEM, n=3. *P<0.05, **P<0.01, ***P<0.01, ****P < 0.0001, as determined by one-way ANOVA.

Data show that HB0043 significantly inhibited IL-6 and IL-8 secretion stimulated with IL-17A and IL-36 in a dose-dependent manner. The inhibition rate of HB0043 was clearly better than that of HB0017 or HB0034 (**Figures 3A–C** and **Supplementary Figure 1B**). Altogether, our data suggest that HB0043 was more efficient than mAbs in the blockade of the secretion of IL-6 and IL-8 induced by IL-17A and IL-36 ligand, thereby may play a better role as a potent dual inhibitor for both IL-17A and IL-36-mediated activities *in vitro*.

### Dual targeting of IL-17A and IL-36R showed improved anti-inflammatory effect in atopic dermatitis model and psoriasis model, compared to single mAbs

**Figure 4:**
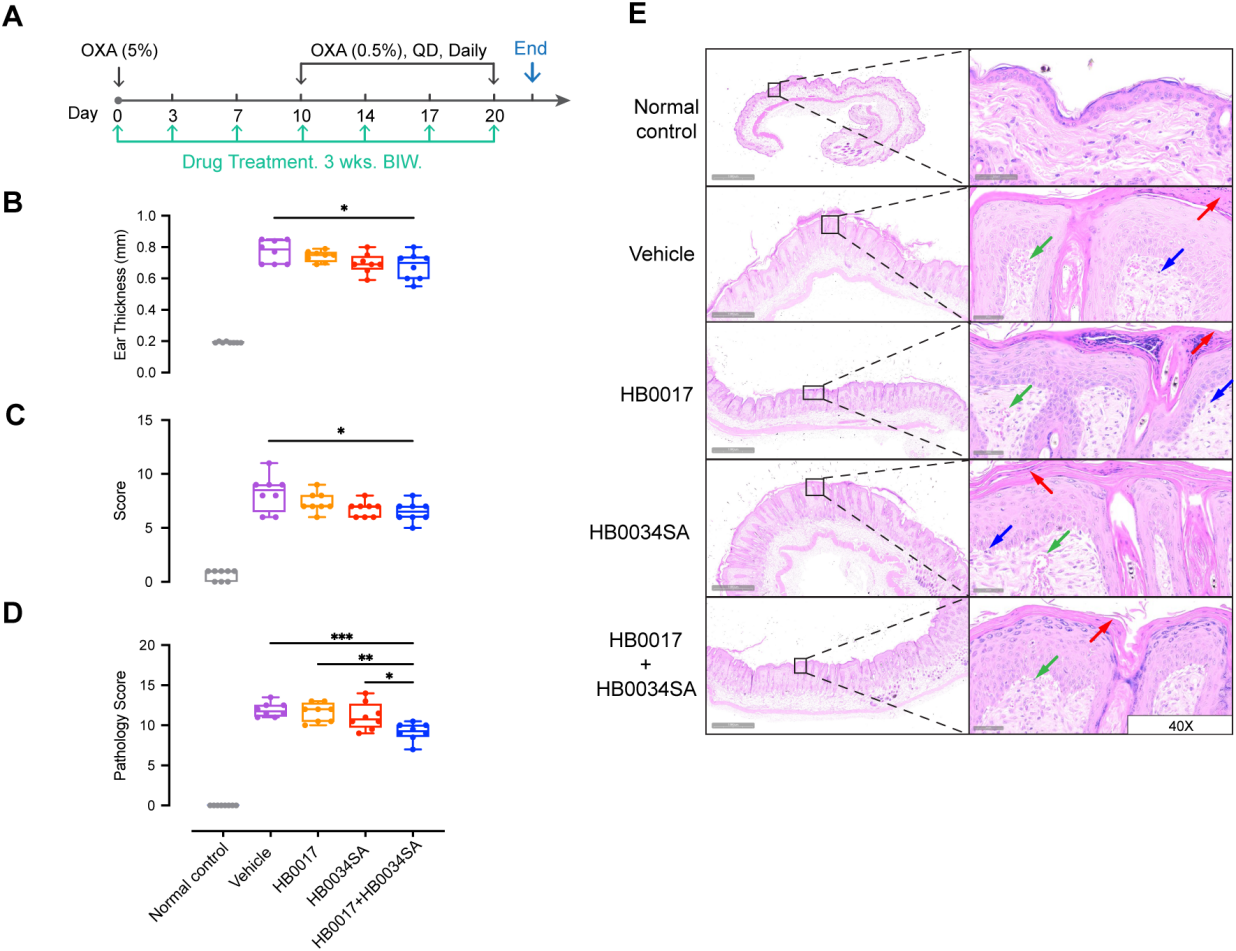
The combination of mAbs targeting IL-17A and IL-36R showed stronger inhibition of inflammation relative to mAbs alone in oxazolone-induced mouse atopic dermatitis model. **(A)** Male C57BL/6J mice were smeared with 5.0% OXA on the shaved back skin and ear on Day0 and followed by a daily topical application of 5.0% OXA on back and 0.5% OXA on ear for thirteen consecutive days from Day7 for acute disease induction. Antibodies (HB0017, HB0034SA, HB0017+HB0034SA) were administered intraperitoneally into mice at 50 mg/kg twice a week. The control group received PBS intraperitoneal injections. **(B, C)** Combination of anti-IL-36R and anti-IL-17A antibodies significantly ameliorated OXA induced increase of ear thickness **(B)** and reduced the clinical score of back skin **(C)**. Clinical score: the comprehensive evaluation of oedema, erythema, dryness and excoriation and each symptom was scored independently on a scale from 0 to 3: 0, none; 1, slight; 2, moderate; 3, severe. Data are expressed as min to max, n=8. *p ≤ 0.05. **(D, E)** Back skin samples were collected for subsequent histopathological evaluation **(D)** and H&E staining **(E)**. Vehicle group: HE staining shows hyperkeratosis, significant thickening of the stratum corneum, acanthosis, and local finger-like protrusions of the epidermis into the dermis. The dermis is edematous, the capillaries are dilated and congested, and accompanied by a small amount of inflammatory cell infiltration. HB0017 and HB0034SA group shows slightly improvement with mild inflammatory cell infiltration, organic lesions in corneum and dermis haven’t shown remission. HB0017+HB0034SA group: Significantly reduced hyperkeratosis and thickening of stratum corneum. Vasodilatation—green arrow; Lymphocytes—blue arrow; Hyperkeratosis/parakeratosis—red arrow. *P<0.05, **P<0.01, ***P<0.01.

Accumulating evidence indicates that the IL-36 agonists and Th17 cytokines have a synergistic pathogenic role in skin inflammation (27, 37, 42). However, it remains to be shown whether co-inhibition of two pathways could provide enhanced alleviation of skin inflammation. Here we investigated the effect of co-inhibition in an oxazolone-induced mouse atopic dermatitis model. Similar to previous studies by others (43), our anti-human IL-36R antibody did not bind to murine IL-36R (**Supplementary Figure 2A** and **Table 2**). For *in vivo* evaluation, we developed a surrogate mAb HB0034SA that bound mouse IL-36R and blocked signaling of mouse IL-36 agonists (**Supplementary Figures 2A, B**). With the HB0034SA and anti-IL-17A antibody HB0017 that cross-reacts to mouse IL-17A (40), we compared the anti-inflammatory effect of combination of two mAbs to single mAbs in oxazolone-induced mouse atopic dermatitis model (**Figure 4A**). When ear thickness was measured, the increase in ear thickness on day 14 in combination group was moderately but significantly smaller compared to the vehicle group (**Figure 4B**). Further, when clinical scores of affected back skin group with combinational treatment had significantly lower scores (**Figure 4C**). When pathological scores based on histological assessment were evaluated, the vehicle group showed hyperkeratosis, thickening of the stratum corneum, acanthosis, and local finger-like protrusions of the epidermis into the dermis. The dermis was edematous, the capillaries were dilated and congested, and accompanied by a small amount of inflammatory cell infiltration (**Figure 4E**). The HB0017 and HB0034SA group showed slight improvement with mild inflammatory cell infiltration. The combination group showed significantly reduced hyperkeratosis and thickening of stratum corneum (**Figure 4D** and **Supplementary Figure 3**).

**Figure 5:**
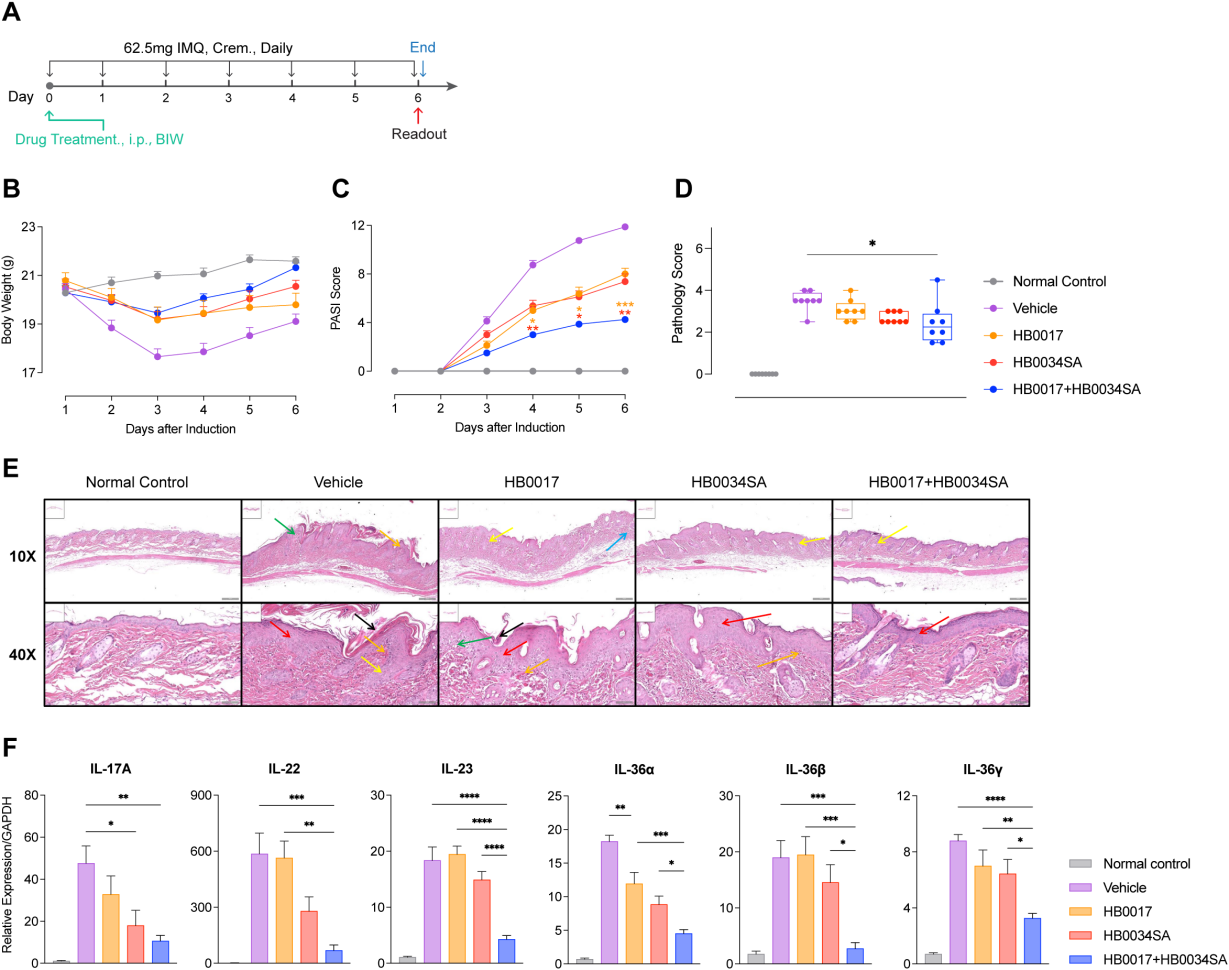
Dual blockade of IL-17A and IL-36R showed stronger inhibition of inflammation relative to mAbs alone in IMQ-induced mouse psoriasis model. **(A)** Schematic plot showing therapeutic dosing regime of BALB/C mice for experimental data in IMQ-induced psoriasis model. Mice were smeared with 5.0% IMQ (62.5mg) on the shaved back skin from Day1 to Day 7 daily. Antibodies (HB0017, HB0034SA, HB0017+HB0034SA) were administered intraperitoneally into mice at 50 mg/kg twice a week. The control group received PBS intraperitoneal injections. **(B)** Body weight. **(C)** PASI score evaluation result, **(D, E)** Back skin samples were collected for subsequent histopathological evaluation **(D)** and H&E staining **(E)** (black arrow: stratum corneum thicken, green arrow: loose structure, red arrow: acanthosis, yellow arrow: trochanterellus lengthen and rodlike, blue arrow: inflammatory cell infiltration, orange arrow: blooding in papillary layer). **(F)** Relative skin mRNA expression of skin inflammation related cytokines (IL-17A, IL-22, IL-23, IL-36α, IL-36β, IL-36γ). *P<0.05, **P<0.01, ***P<0.01, ****P<0.0001.

Adding evidence to mouse atopic dermatitis model, results of mouse IMQ-induced acute psoriasis model kept showing the better efficacy of dual-blocking IL-17A and IL-36R (**Figure 5**). In this acute model, antibodies were given at the beginning of IMQ treatment (**Figure 5A**). While IMQ treatment caused loss of body weight, antibody-treated group did not have overt loss of body weight (**Figure 5B**). PASI score of skin inflammation (**Figure 5C**), combination of HB0017 and HB0034SA had better alleviation of skin inflammation (based on PASI score and histopathologic evaluation) than HB0017 and HB0034SA. (**Figures 5D, E**). Further, combination of HB0017 and HB0034SA suppressed more potently the mRNA expression of inflammatory cytokines in the lesions (**Figure 5F**). Together, *in vivo* data support that dual blockade of IL-17A and IL-36R had improved efficacy in suppressing skin inflammation.

### HB0043 had no overt increase in systemic toxicity but had higher rate of anti-drug antibodies, compared to mAbs in cynomolgus monkeys

For toxicity evaluation, HB0034 (at doses of 50, 100, or 250 mg/kg) and HB0043 (at doses of 20, 100, or 200 mg/kg) were given to cynomolgus monkeys by intravenous infusion. HB0017 was given to cynomolgus monkeys by subcutaneous injection at doses of 15, 50 or 150 mg/kg. Abs were given once a week, 5 times in total, and followed by a 6-week recovery. The NOAEL (No Observed Adverse Effect Level) of HB0017, HB0034 and HB0043 were determined as 150 mg/kg, 250 mg/kg, 200 mg/kg respectively and no severe target-related toxicity was found (**Table 3**). One of adverse reactions was skin erythema, which normally disappeared within 3 days after administration. Mild to moderate subcutaneous inflammation could be seen in the local area of administration in all groups. During the experiment, other clinical parameters including body temperature, electrocardiogram, coagulation function, lymphocyte subsets and urinalysis showed no significant changes related to HB0017, HB0034 and HB0043 (**Table 3**).

**Table 3:** Nonclinical safety evaluation in cynomolgus monkeys.

	HB0017	HB0034	HB0043*
Animal species	Cynomolgus monkey	Cynomolgus monkey	Cynomolgus monkey
Route of administration	Subcutaneous	Intravenous	Intravenous
Dosage (mg/kg)	15, 50, 150	50, 100, 250	20, 100, 200
Dosing cycle	Once a week, a total of 5 doses	Once a week, a total of 5 doses	Once a week, a total of 5 doses
NOAEL	150 mg/kg	250 mg/kg	200 mg/kg
Main toxicological findings	IgG level elevated and local inflammation and/or hemorrhage observed.	Total protein, globulin concentration, and IgG occasionally increased.	After the 3^rd^ and 5^th^ dosage, 200 mg/kg group GLB increased, A/G decreased; 200 mg/kg group males TP increased, IgG increased in 100, 200 mg/kg groups.

In cynomolgus monkeys, following a single *s.c.* dose of 3~30 mg/kg HB0017, a single *i.v.* dose of 5~50 mg/kg HB0034, or a single *i.v.* dose of 3~30 mg/kg HB0034, approximately linear kinetics were observed for all the three Abs. The systemic exposure (AUC) showed no significant difference among the three Abs (**Table 4**). HB0043 exhibited a shorter half-life (5.8 ± 2.4 ~ 7.1 ± 2.2 days) compared to HB0017 (6.2 ± 4.2 ~ 11.2 ± 1.3 days), but comparative to HB0034 (5.2 ± 2 ~ 6.7 ± 1.9 days), indicating a potential long dosing interval for the HB0043 in humans (**Table 4** and **Supplementary Figure 4**). Anti-drug antibodies (ADA) were detected after administration of the three Abs, with a higher prevalence observed in HB0043. However, no apparent influence was noted on blood drug concentration and PK parameters calculation, including t_1/2._ Furthermore, no systemic toxic effects induced by ADA were observed.

**Table 4:** Single-dose pharmacokinetic study in cynomolgus monkeys.

	HB0017	HB0034	HB0043
Animal species	Cynomolgus monkey	Cynomolgus monkey	Cynomolgus monkey
Administration	*s.c.*	*i.v.*	*i.v.*
Dosage cycle (mg/kg)	3, 10, 30	5, 15, 50	3, 10, 30
AUC_(0~t)_ (h*mg/mL)	18.8 ( ± 2.1) ~191.4 ( ± 37.9)	14.5 ( ± 2.4) ~159.4 ( ± 32.6)	13.2 ( ± 3.3) ~169.6 ( ± 39.6)
C_max_ (μg/mL)	48.1 ( ± 6.6) ~522.7 ( ± 55.1)	158.0 ( ± 17.9) ~1579.3 ( ± 332.7)	99.9 ( ± 13.6) ~1143.7 ( ± 173.5)
t_1/2_ (h)	149.7 ( ± 99.7) ~268.8 ( ± 31.2)	124.8 ( ± 48.0) ~160.8 ( ± 45.6)	138.9 ( ± 58.0) ~170.4 ( ± 53.0)
ADA detection rate	0~33.3%	0~67%	83.8%~100%

## Discussion

IL-17 inhibitors have greatly advanced the treatment for plaque psoriasis and psoriatic arthritis (44). More recently, IL-36 inhibition has also been shown to have remarkable therapeutic effect on generalized pustular psoriasis (GPP), a severe neutrophilic skin disease (45). Our own anti-IL-36R Ab HB0034 has also entered clinical trials for normal subjects and GPP patients [NCT05064345, NCT05512598, NCT06231381, NCT06477536]. Nevertheless, challenges remain for treatment of chronic inflammatory skin diseases (46). Evidence over the years has shown IL-17 and IL-36 cooperate to drive skin inflammation (27, 37, 38, 42, 44, 45), however there is no therapeutic agent currently available targeting both IL-17 and IL-36. Here we demonstrated that the dual blockade of IL-17A and IL-36R enhanced alleviation of skin inflammatory responses, based on *in vitro* and *in vivo* data. These data provide some insights how interplay of two cytokines can be targeted.

Cooperation of IL-17 and IL-36 in skin inflammation can operate through several mechanisms. It is known that Th17 cytokines can induce IL-36 production by keratinocytes; in turn, IL-36 cytokines synergize with IL-17A and TNF-α to increase their own expression in keratinocytes (27, 37). IL-36 then stimulates production of proinflammatory cytokines TNF-α, IL-6 and IL-8 by keratinocytes, and IL-17 could provide synergy to IL-36 to induce these proinflammatory cytokines (27). Apart from keratinocytes, IL-36 also activates dendritic cells/macrophages to amplify skin inflammation (46). Further, IL-36 regulates the recruitment of inflammatory cells and the expansion of IL-17A–producing γδ T cells in the skin (45). Here we probed the cooperative action of IL-36 and IL-17 in human dermal fibroblasts (NHDFs), another major skin resident cells. Similar to what has been described for keratinocytes (38), an IL-17A and IL-36 combination induced many differentially expressed genes including those encoding cytokines, chemokines and fibrosis-related genes matrix metalloproteinases in NHDFs. Thus, these two cytokines could directly act on fibroblasts to amplify inflammatory and fibrotic responses.

Although above evidence indicated that IL-17 and IL-36 can cooperate in multiple cell types, it remains to be established whether dual targeting could enhance the alleviation of skin inflammation. *In vitro*, we found that BsAb HB0043 could inhibit the production of IL-6 and IL-8 by NHDFs, more potently than single mAbs, supporting dual target therapy in difficult-to-treat skin inflammation. In an oxazolone-induced mouse atopic dermatitis model and IMQ-induced skin inflammation, we showed the enhanced anti-inflammatory effect of dual targeting of IL-17A and IL-36R employing a surrogate anti-mouse IL-36R mAb HB0034A and a mouse cross-reactive anti-human IL17A mAb HB0017, compared to single mAbs. Thus, the dual blockade of IL-17A and IL-36R may broaden the treatment for chronic inflammatory skin diseases, as well as chronic inflammatory diseases of other tissues.

Dual biologic (antibody) therapy has now extended to diseases beyond cancers (47, 48). For autoimmune and inflammatory diseases, dual biologic therapy mainly targets cytokines, chemokines and immune cells (47, 48). Conceivably, dual biologic therapy comes in several forms: administration of two or more mAbs, administration of antibody mixtures (49) and administration of BsAbs (50). Each form has its own advantages and limitations. A clear advantage for BsAbs, as a single modality, is the reduced cost of drug development and clinical application. Further, if the one target of BsAbs is a cytokine receptor, such BsAbs could perceivably increase local drug availability for other target. Limitations for development of BsAbs include manufacturing challenges, reduced half-life and increased production of anti-drug antibody (ADA) (38). Nevertheless, our BsAb HB0043 largely maintained the functionalities of parental mAbs. In cynomolgus monkeys, HB0043 engineering did not impair the half-life compared to the parental antibody HB0017 and HB0034. Thus, HB0043 has a relatively preserved half-life compared to mAbs. We did observe higher occurrence of ADA, however, it did not drastically affected drug distribution. While increased toxicity is a drawback for certain dual biologic (antibody) therapies (51), HB0043 did not display overt increase in toxicity compared to two parental mAbs in a study in cynomolgus monkeys. Thus, we predict that dual targeting IL-17 and IL-36 would present a favorable safety profile. However, the preclinical evaluation of toxicity is often performed in very confined facility, it needs to be cautious that patients in real world may face adverse effects like increased risk of infection.

**Figure 6:**
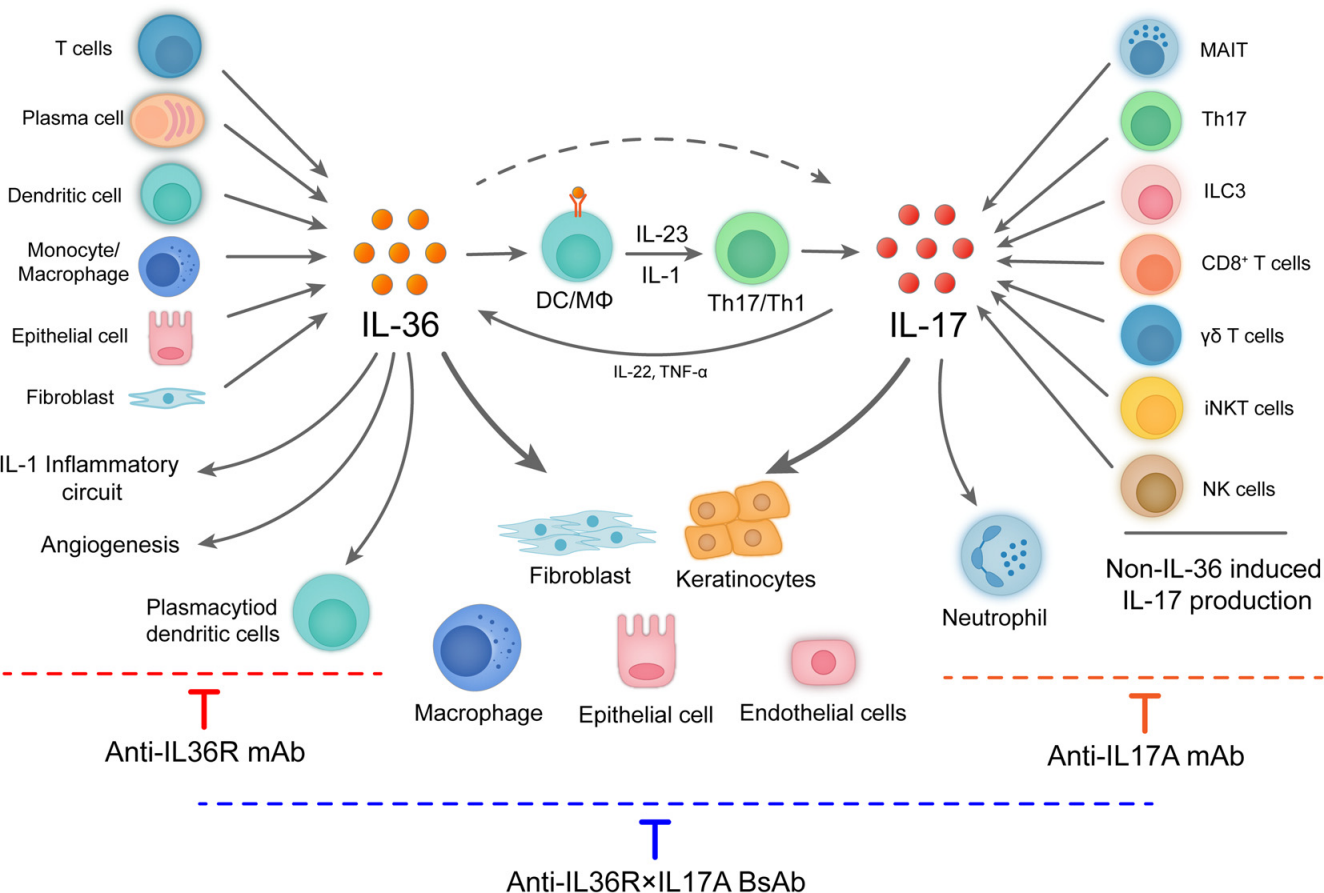
IL-36 and IL-17 secreted by multiple cell types have overlapping and non-overlapping downstream effects. IL-36 cytokines are expressed by a broad variety of cell types such as epithelial cell, fibroblast, keratinocytes, lymphocytes, monocytes, myeloid dendritic cells (DCs), monocyte-derived DCs, plasmacytoid DCs, and plasma cells. There is a feedback loop between the IL-36 and Th17 cytokines. Indeed, IL-36 cytokines are not only regulated by Th17 cytokines, but also act as activators of Th17 cells which regulate the differentiation and enhance the expression of Th17 cytokines. IL-17 is mainly secreted by IL-23-IL-17 axis, as well as other cell populations, such as CD8+ (Tc17) cells and various subsets of innate lymphocytes including γδT cells, natural killer T (NKT) cells, group 3 innate lymphoid cells (ILC3s), and ‘‘natural’’ Th17 cells. IL-36 and IL-17 signaling promote secretion of inflammatory and chemotactic molecules and immune cell infiltration and immune regulation, which closely related to many inflammatory diseases.

As we are potentially approaching the therapeutic ceiling of monotherapy in many autoimmune and inflammatory diseases as illustrated for IBD (51), the development of rational combination therapies including dual biologic therapy may be important to improve disease control. With clear understanding of synergistical model of action, we believe that dual biologic therapy could broaden the treatment for the diseases that have not benefit optimally from mono-targeting. Dual biologic therapy could either increase the therapeutic efficacy of biologics that has shown to be effective but not optimal or render effective to indications that monotherapy is not effective. As our study provided a proof-in-concept of dual targeting the overlapping and non-overlapping actions IL-36R and IL-17A in preclinical studies (**Figure 6**), with a focusing on skin inflammation, we anticipate that dual blockade of IL-17A and IL-36R have potentials in many other inflammatory/fibrotic diseases with the involvement of these two inflammatory cytokines. One example of such diseases is hidradenitis suppurativa (HS). Both IL-17 and IL-36R had been targeted in clinical studies (52). As targeting IL-36R alone may have limited efficacy as reported for NCT04856930, it may have potential to increase the efficacy of IL-17 targeting in this disease. Beyond targeting IL17A and IL-36R, rational design of combination therapies targeting other critical players of inflammatory/fibrotic diseases could provide a new revenue for treatment of diseases with unmet medical needs. Beyond, skin inflammation, IL-17 antagonism has been investigated for lupus nephritis with limited successes (53). Given that IL-36 has been implicated in lupus nephritis (54), co-targeting both IL-17 and IL-36R may also increase therapeutic potentials. Overall, development of dual target biologics has gained momentum to overcome therapeutic celling of monotherapy. Anti-TSLP/anti-IL-13 biologic nanobody improved lung function and small airway dysfunction in asthma beyond those reported for monovalent IL-13 or TSLP pathway targeting (55). Finally, although dual targeting in treatment of inflammatory and autoimmune diseases shows great promise, evident from preclinical studies and some early clinical studies, it remains to find out how to realize these promises to actual clinical benefit, considering that difficult-to-treat diseases often have complex and even undefined pathogenesis.

## Data Availability

The data presented in the study are deposited in the NCBI database, accession number is PRJNA1181153, http://www.ncbi.nlm.nih.gov/bioproject/1181153.
