# Supplementary material for: Dual blockade of IL-17A and IL-36 pathways via a bispecific antibody exhibits enhanced anti-inflammatory potency
Source: Front Immunol. 2024 Nov 12;15:1434127. doi: 10.3389/fimmu.2024.1434127 (PMC11590123; doi:10.3389/fimmu.2024.1434127)
Supplement: Supplementary file 1 [file DataSheet1.docx]

# Supplementary Figures


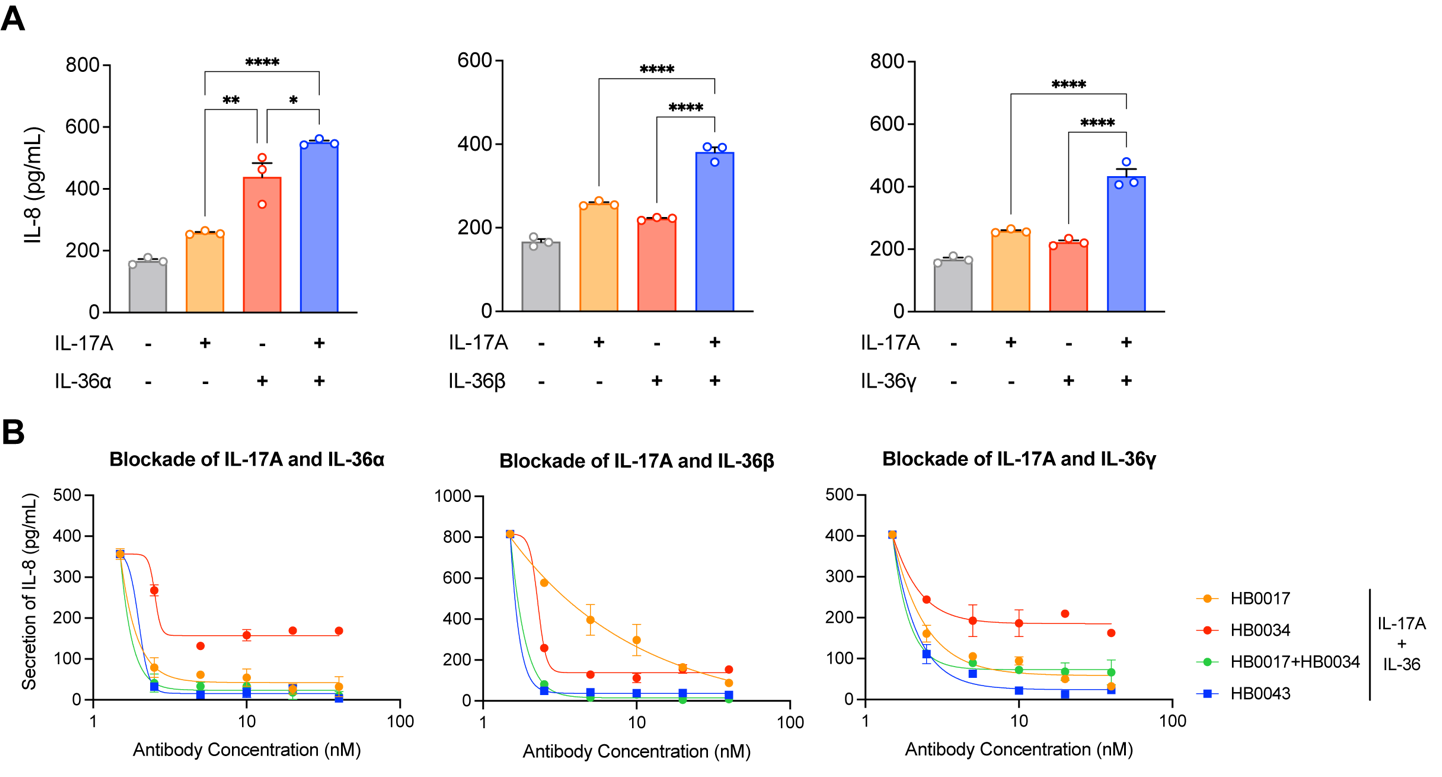


**Supplementary Figure 1. HB0043 inhibited IL-8 secretion stimulated by IL-17A and IL-36 in NHDF.**

**(A**) The effect of IL-17A and IL-36 ligand in the induction of IL-8 secretion. NHDF cells were cultured with 0.5 ng/ml IL-17A and 15 ng/ml IL-36α, 1 ng/ml IL-36 β or 2 ng/ml IL-36 γ overnight. **(B)** The ability of HB0043 to block IL-36R signaling and IL-17A signaling was measured and compared to that of HB0034 and HB0017, using IL-17A and IL-36R blockade cell-based assay. The secretion of IL-8 was monitored, which was induced by 0.5 ng/ml human IL-17A and 15 ng/ml IL-36 α, 1 ng/ml IL-36 β or 2 ng/ml IL-36 γ in NHDF cells overnight. Data are expressed as means ± SEM, n=3. ****P ≤ 0.0001, as determined by one-way ANOVA.


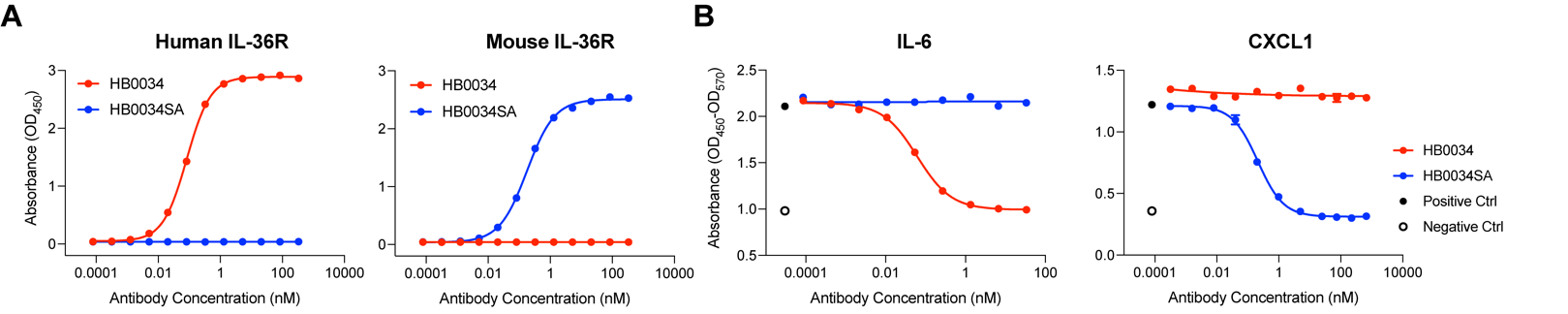


**Supplementary Figure 2. Functional assay of generated mouse IL-36R-targeted antibody**

**(A)** ELISA array, HB0034 binds to human IL-36R with affinity of 0.083nM but not mouse IL-36R. Therefore, generated mouse-targeted mAb HB0034SA can bind mouse IL-36R with affinity of 0.18 nM; **(B)** HB0034 can inhibit IL-6 expression in NCI/ADR-RES cells at 0.061 nM, HB0034SA also block the CXCL1 in NIH/3T3 cells at 0.21 nM;

**
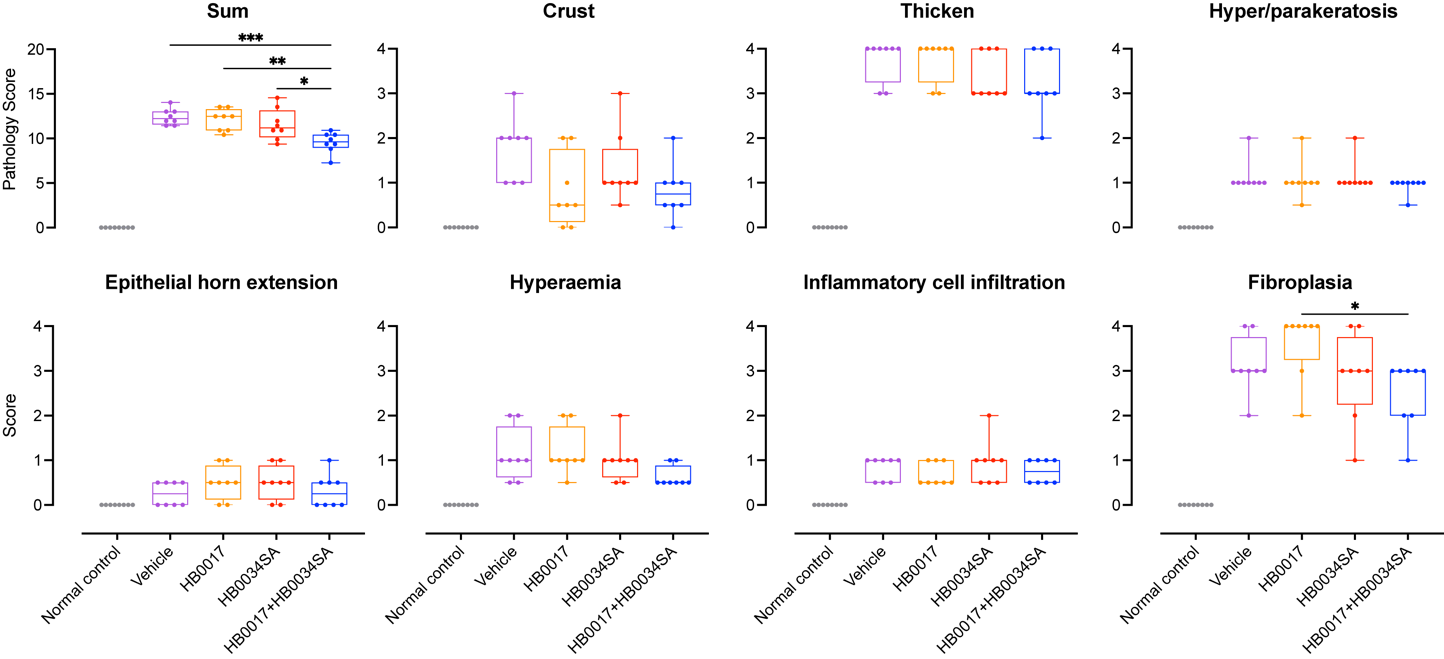
**

**Supplementary Figure 3. Back skin pathology scoring of oxazolone-induced mouse atopic dermatitis model.**

Pathological score: the decrustation, [incrassation](javascript:;), hyperkeratosis or parakeratosis, epithelial horn extension, [hyperaemia](javascript:;), inflammatory cell infiltration and fibrogenesis were evaluated according to the 5-level scoring method: 0. normal appearance; 0.5. sporadic or slight changes compared with normal appearance; 1. minor changes; 2. moderate changes; 3. severe changes; 4. extraordinary serious changes in tissues or cells numbers associated with the development of disease. Arrows in H&E staining of representative pictures: Vasodilatation--green; Lymphocytes--blue; Hyperkeratosis/parakeratosis--red. Data are expressed as min to max, n=8. *P≤0.05, **P≤0.01, ***P≤0.001, as determined by one-way ANOVA.


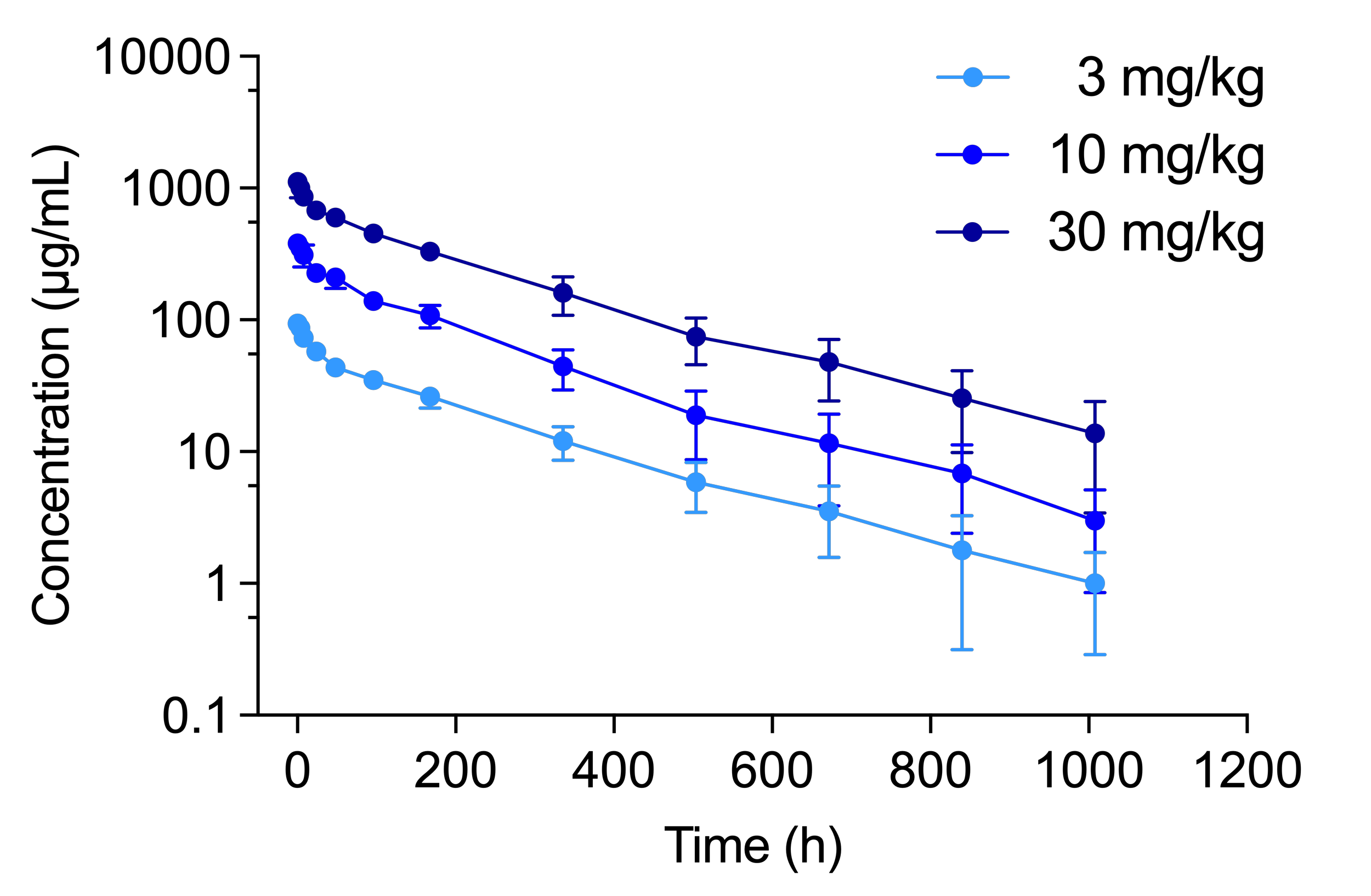


**Supplementary Figure 4. HB0043 showed linear pharmacokinetic characteristics**

The pharmacokinetic profiles of HB0043 in cynomolgus monkeys at doses 3, 10, and 30 mg/kg were shown. data expressed as mean ± SEM.
